# An innovative strategy for standardized, structured, and interoperable results in ophthalmic examinations

**DOI:** 10.1186/s12911-020-01370-0

**Published:** 2021-01-06

**Authors:** Yongseok Mun, Jooyoung Kim, Kyoung Jin Noh, Soochahn Lee, Seok Kim, Soyoung Yi, Kyu Hyung Park, Sooyoung Yoo, Dong Jin Chang, Sang Jun Park

**Affiliations:** 1 Department of Ophthalmology, Seoul National University College of Medicine, Seoul National University Bundang Hospital, 82, Gumi-ro 173 Beon-gil, Bundang-gu, Seongnam-si, Gyunggi-do 13620 Republic of Korea; 2 School of Electrical Engineering, Kookmin University, 77, Jeongneung-ro, Seongbuk-gu, Seoul Republic of Korea; 3 Healthcare ICT Research Center, Office of eHealth Research and Businesses, Seoul National University Bundang Hospital, 172, Dolma-ro, Bundang-gu, Seongnam-si, 13605 Gyunggi-do Republic of Korea; 4 Department of Ophthalmology, College of medicine, The Catholic University of Korea, Yeouido St. Mary’s Hospital, 10, 63-ro, Seoul, 07345 Yeongdeungpo-gu Republic of Korea

**Keywords:** Optical coherence tomography, Optical character recognition, Deep learning, Text detection

## Abstract

**Background:**

Although ophthalmic devices have made remarkable progress and are widely used, most lack standardization of both image review and results reporting systems, making interoperability unachievable. We developed and validated new software for extracting, transforming, and storing information from report images produced by ophthalmic examination devices to generate standardized, structured, and interoperable information to assist ophthalmologists in eye clinics.

**Results:**

We selected report images derived from optical coherence tomography (OCT). The new software consists of three parts: (1) The Area Explorer, which determines whether the designated area in the configuration file contains numeric values or tomographic images; (2) The Value Reader, which converts images to text according to ophthalmic measurements; and (3) The Finding Classifier, which classifies pathologic findings from tomographic images included in the report. After assessment of Value Reader accuracy by human experts, all report images were converted and stored in a database. We applied the Value Reader, which achieved 99.67% accuracy, to a total of 433,175 OCT report images acquired in a single tertiary hospital from 07/04/2006 to 08/31/2019. The Finding Classifier provided pathologic findings (e.g., macular edema and subretinal fluid) and disease activity. Patient longitudinal data could be easily reviewed to document changes in measurements over time. The final results were loaded into a common data model (CDM), and the cropped tomographic images were loaded into the Picture Archive Communication System.

**Conclusions:**

The newly developed software extracts valuable information from OCT images and may be extended to other types of report image files produced by medical devices. Furthermore, powerful databases such as the CDM may be implemented or augmented by adding the information captured through our program.

## Background

Ophthalmic examinations have made remarkable progress in recent years. In clinical practice, ophthalmic examinations are necessarily aided by specific technologies, including optical coherence tomography, ophthalmic biometry, corneal topography, and others. Although these technologies and devices are widely used, they produce different standardized image formats, such as the digital imaging and communications in medicine (DICOM) standard, and also other standardized result reporting formats [1]. In most cases, ophthalmologists should use the dedicated viewer provided by each device and manufacturer to read the captured images, and may review the resulting reports in portable document format (PDF), or review the images directly. Most ophthalmic devices do not provide a way to access internal data directly and lack standardization in both image review and results reporting systems, making interoperability unfeasible. In addition, the lack of standardization also makes it difficult for ophthalmologists to observe overall improvement or progression trends, since the reports are not structured to enable longitudinal data assessment. From the clinician’s point of view, the first and most important step is to organize results in a structured and standardized format, with subsequent storage of the information in a clinician-friendly and interoperable system. If recently developed computer vision and deep-learning technologies are coupled with optical character recognition (OCR), then this medical need may be addressed by enabling the creation of software customized for reading reports produced by ophthalmic examination devices. This effort is specifically required because existing legacy software cannot be customized. In addition, deep-learning-based algorithms could also provide reliable classification (e.g., disease presence), or segmentation results (e.g., amount of fluid indicating disease severity) from ophthalmic images [2–5]. Consequently, we developed and validated new software designed to extract, transform, and store data from report images produced by ophthalmic examination devices, providing ophthalmologists in eye clinics ready access to standardized, structured, and interoperable information.

## Implementation

Among the various types of equipment available to ophthalmologists, optical coherence tomography (OCT) is crucial for non-invasive clinical evaluation of the optic disc and retina in various ophthalmic diseases. OCT produces tomographic images of retinal optical reflectivity analogous to an ultrasound B-scan, but with much higher resolution [6]. Spectrum-domain OCT, or swept-source OCT, which has a higher resolution than conventional time-domain OCT, can provide highly-detailed images of the structure of each retinal layer [7]. In fact, present day ophthalmologists in clinical practice could not possibly provide a definitive diagnosis without OCT. The OCT technology enables quantification of retinal structures that must be evaluated such as the optic disc, retinal nerve fiber layer, and macula, since they can be a major cause of blindness [8]. At the Seoul National University Bundang Hospital, the number of OCT tests performed in one year reached 54,041 in 2018 and continues to increase rapidly. All these test results are stored only as image files in the hospital Electric Health Record (EHR) system.

We have developed new software to extract measurement values from Spectralis OCT report images (Heidelberg engineering, Heidelberg, Germany), and import them into a well-defined database. The new software can also import various reports derived from other ophthalmic examination devices by modifying configuration files containing the coordinates of image regions of interest. All of our software source code is freely available on the GitHub. (https://github.com/ophthal-cdm/SNUBH_CharReadOCT).

**Fig. 1:**
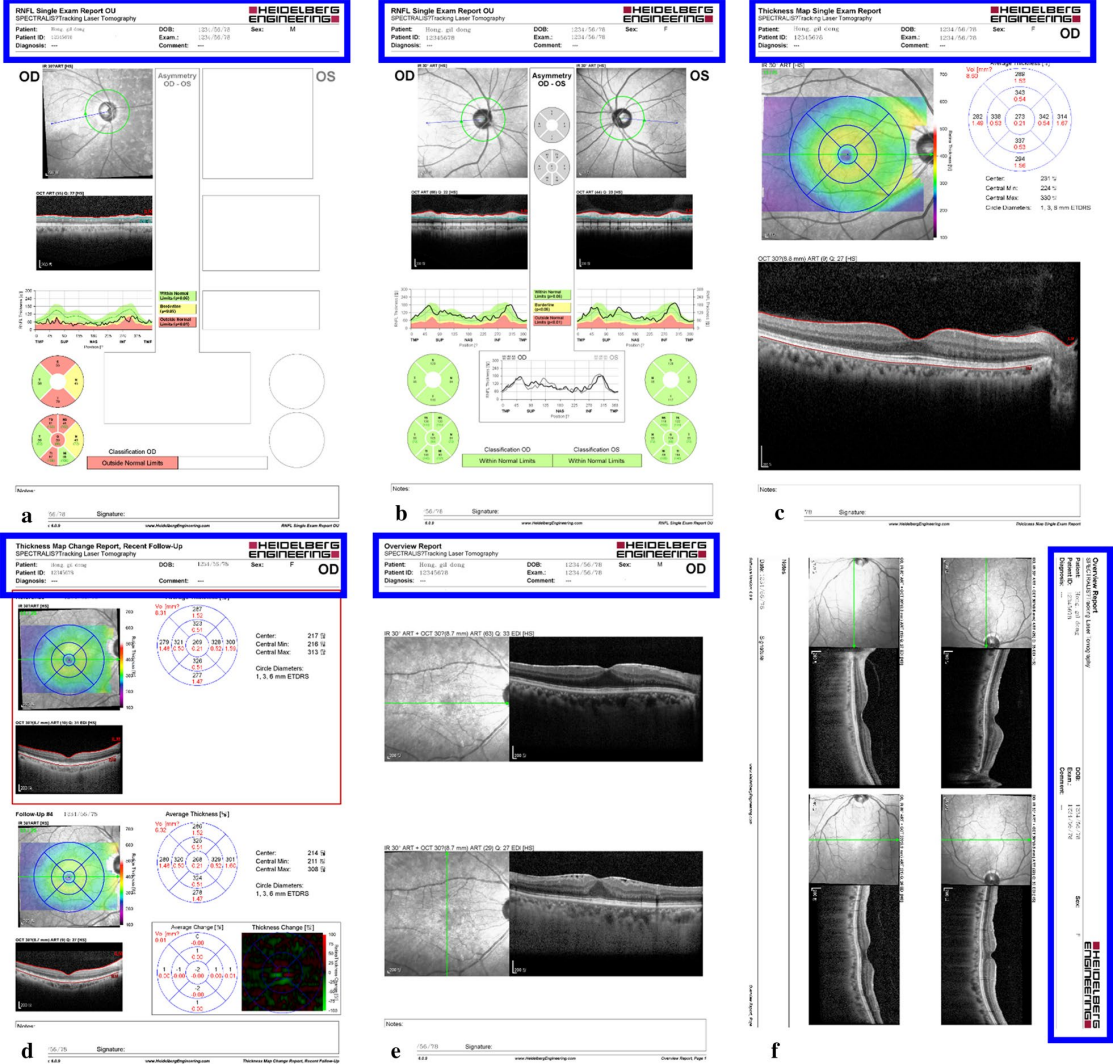
Examples of reports including important information provided by the software. **a** unilateral RNFL thickness; **b** bilateral retinal nerve fiber layer (RNFL) thickness; **c** unilateral macula thickness and volume, current only; **d** unilateral macula thickness and volume, previous and current; **e** unilateral horizontal and vertical macula scan, current only; **f** unilateral horizontal and vertical macula scan, previous and current. The blue boxes indicate the area read by the classifier. The titles at top left differ from one another

**Fig. 2:**
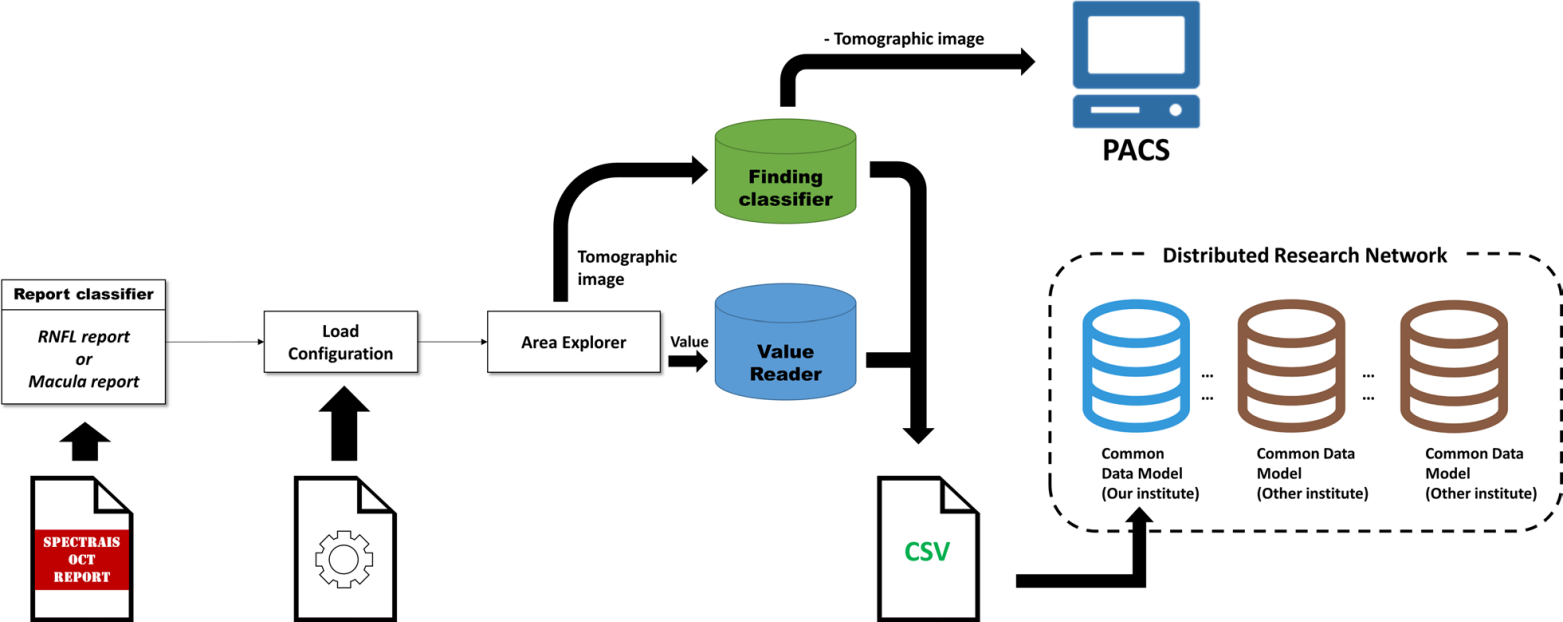
Overall schematic diagram of our software. *CSV* comma-separated value; *PACS* picture archiving communication system

**Fig. 3:**
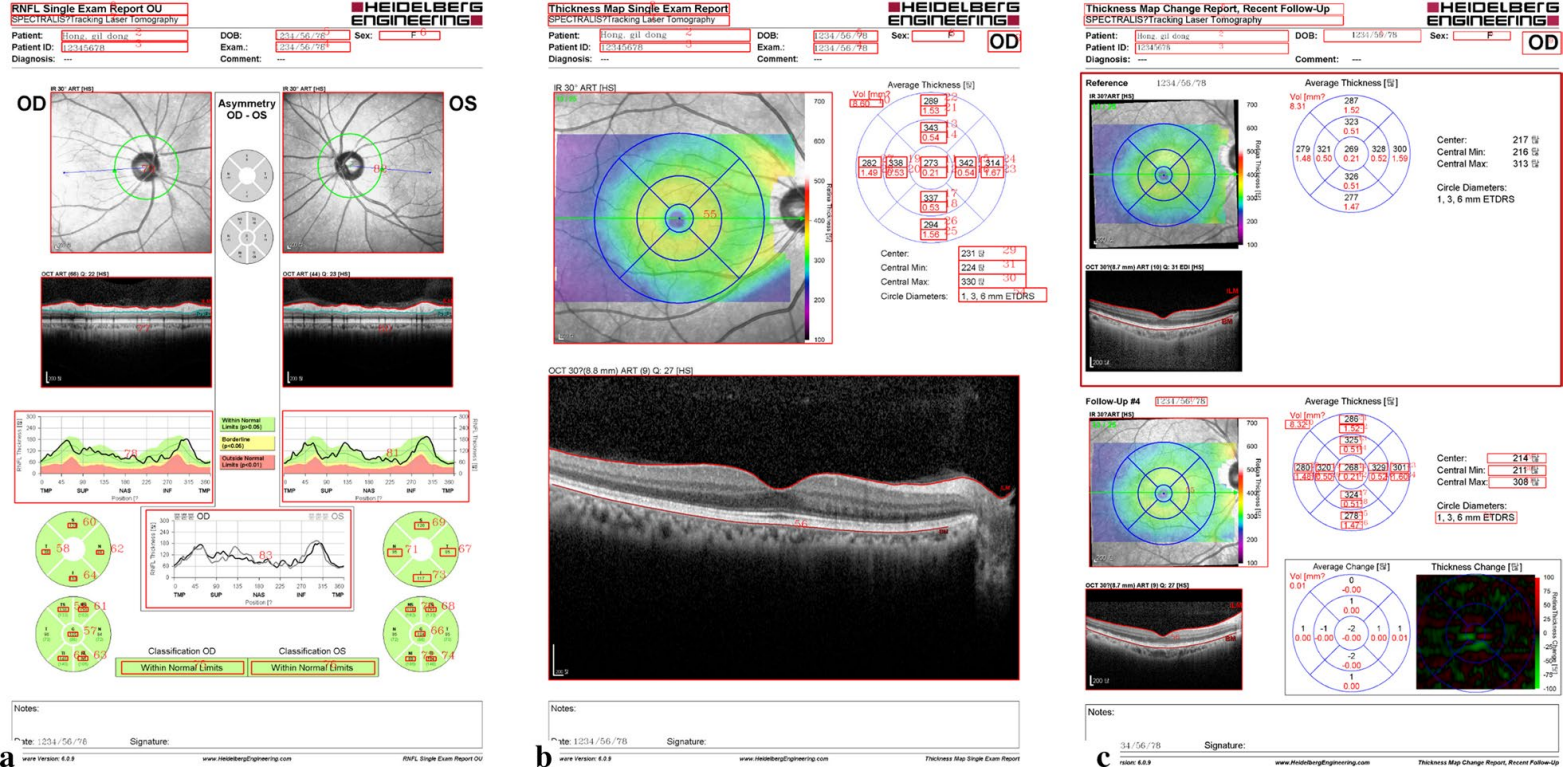
RNFL and macula reports. Red boxes indicate the area selected for recognition

Six report types were found across all versions of Spectralis OCT (from 1.7.0.0 to 1.10.4.0). They were processed as follows (Fig. 1): (1) unilateral retinal nerve fiber layer (RNFL) thickness; (2) bilateral RNFL thickness; (3) unilateral macula thickness and volume, current only; (4) unilateral macula thickness and volume, previous and current; (5) unilateral horizontal and vertical macula scan, current only; and (6) unilateral horizontal and vertical macula scan, previous and current. The first four reports include numeric values that are converted to text, whereas the second two reports only include tomographic images. We used Python with open-source libraries including Tensorflow, Numpy, OpenCV, Xlswriter, and argparse. We summarized how our software extracts values and classifies tomographic image from OCT report images in Fig. 2. Initially, the report classifier identifies the type of report according to the header (Fig. 1). The configuration file determines the location of each numeric value according to the report type. The area of interest using these coordinates is selected (Fig. 3). If the area contains numeric values, Area Explorer delivers it to the Value Reader module, which converts the numeric values to text. If the area contains a tomographic image, Area Explorer delivers it to Finding Classifier, a deep learning-based algorithm which classifies the pathological findings in tomographic images (Fig. 1e, f).

**Fig. 4:**
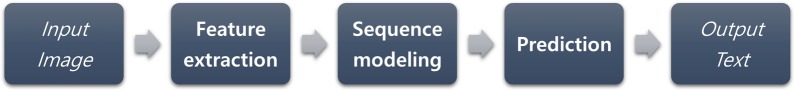
Schematic diagram of Value Reader

**Fig. 5:**
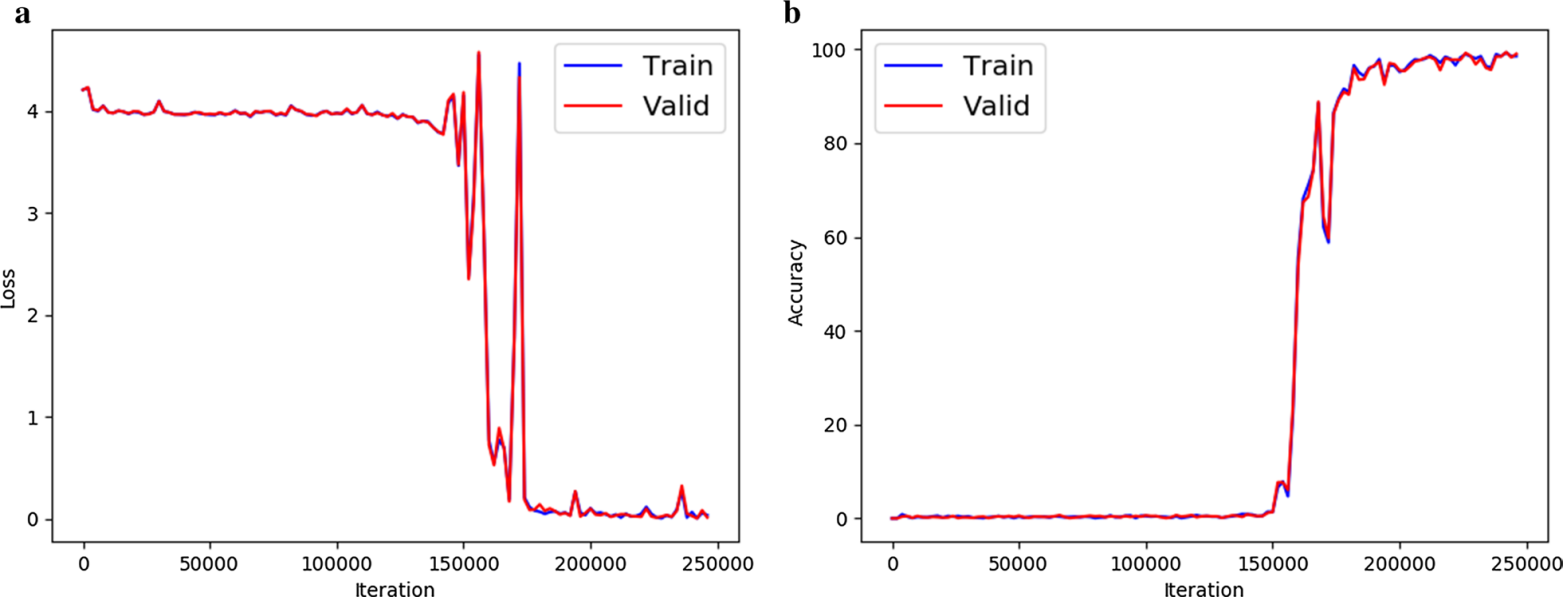
Learning curves of **a** loss and **b** accuracy of Value Reader

**Fig. 6:**
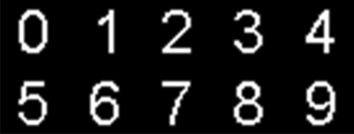
Samples of synthetic data

The Value Reader and Finding Classifier modules are based on a deep-learning algorithm, since previously developed OCR libraries demonstrated poor recognition accuracy for numeric values in OCT report images. The Value Reader module was based on novel methodology for scene text recognition introduced by Baek et al. [9] and an overview is shown in Fig. 4. The feature extraction stage maps the input image to a representation focused on the attributes for character recognition. This stage handles invariant features such as contrast, color, size, and background. We used ResNet in the feature extraction stage to prevent features (gradient) vanishing as the convolutional layers become deeper [10]. The sequence modeling stage captures the contextual information within a sequence of characters [9]. This stage processes visual features from the feature extraction stage, similar to reading a book from left to right, and bidirectional long short-term memory is used to prevent gradient vanishing during backpropagation. [11] The prediction stage estimates the output character sequence [9]. In this stage, characters are generated recurrently from the contextual features obtained from the sequence modeling stage [12]. In our study, the sentence length that could be recognized at one time was limited to 50 characters. The training batch size was 32, and the number of iterations was 160,000. The AdaDelta optimizer (learning rate = 1, decay rate = 0.95) was used [9, 13]. Learning curves of loss and the Value Reader accuracy are shown in Fig. 5. We synthesized datasets to train Value Reader using Text Recognition Data Generator [14]. This approach enabled generation of huge combinations of numbers and characters with difference sizes, contrasts, and amounts of background noise (Fig. 6). We used approximately one million samples of synthetic data for training (0.92 million) and validation (0.08 million). The Finding Classifier was implemented in the same manner as previously reported. [2]

Finally, all processed data were stored in pre-defined comma-separated values (CSV) files and subsequently loaded into a database, such as the Observational Medical Outcomes Partnership (OMOP) common data model (CDM). In addition, the cropped tomographic images were stored in the Picture Archiving and Communication System (PACS) as described in Fig. 2. In order to validate the Value Reader, 300 OCT report images were randomly selected and labeled separately by two experts. The accuracy was assessed by determining whether the expert’s results matched the Value Reader labels. After assessment, the complete set of stored report images was imported into the database.

## Results

**Fig. 7:**
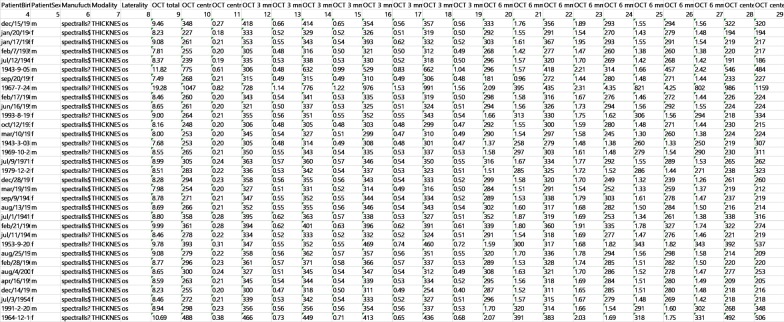
Content of a comma-separated value file produced by Value Reader and finding classifier module. Longitudinal data of each patient can be presented easily by simple manipulation such as sorting or filtering

**Fig. 8:**
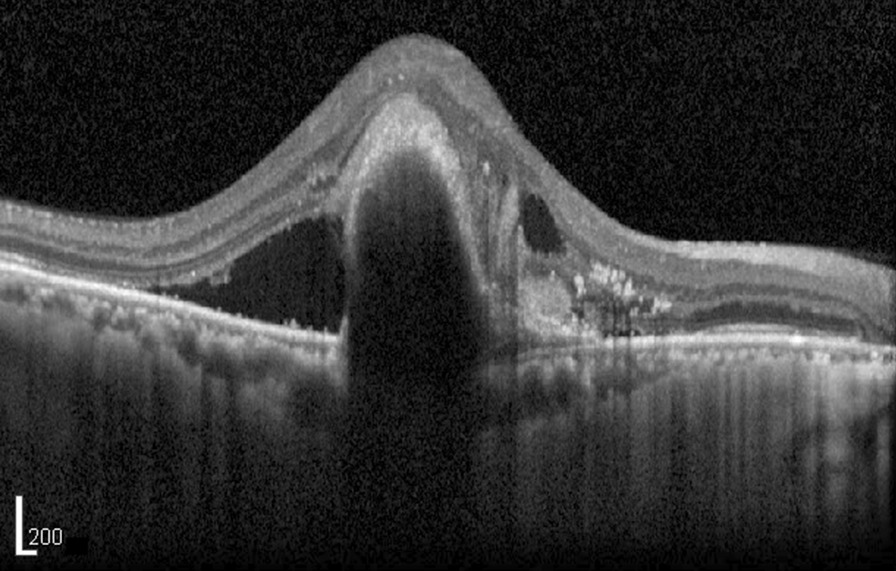
An example of a tomographic image

We extracted a total of 433,175 OCT report images from the Spectralis OCT stored in the hospital EHR system from 7/4/2006 to 8/31/2019. The algorithms used for the unilateral and bilateral RNFL reports were identical since they had the same format. Two report types (Fig. 1e, f) containing only tomographic images without characters were the Finding Classifier module targets, and therefore the accuracy of only three report types was validated. The Value Reader achieved an overall accuracy of 99.67%. Table 1 shows the accuracy of the Value Reader module for each report. The longitudinal dataset from each patient was easily viewed by sorting or filtering (Fig. 7), enabling straightforward recognition of changes and overall trends in measurements. The Finding Classifier accuracy was beyond the scope of our study, but a representative tomographic image is shown in Fig. 8. The presence of subretinal fluid or macular edema reflecting disease activity was indicated by the Finding Classifier to support decisions made by the physician, and the results in CSV file format were successfully loaded into our hospital OMOP CDM. Cropped tomographic images were stored separately and will be loaded into the PACS as soon as possible.

**Table 1:** Value Reader module accuracy as evaluated by two human experts

	Total	RNFL	Macula: current only	Macula: previous and current
By Expert 1				
Number of reports	300	100	100	100
Success (%)	99.67%	100%	99%	100%
Failure (%)	0.33%	0%	1%	0%
By Expert 2				
Number of reports	300	100	100	100
Success (%)	99.67%	100%	99%	100%
Failure (%)	0.33%	0%	1%	0%

An example of how to apply the new software in clinical practice follows. For instance, if a patient is diagnosed with diabetic macular edema requiring intravitreal anti-vascular endothelial or steroid injections, the patient’s retina should be evaluated at every visit by OCT. The Value Reader module displays retinal thickness changes numerically, and the Finding Classifier indicates the presence of macular edema and disease activity (Table 2). Therefore, clinicians receive valuable evidence from these structured data to inform diagnostic decisions.

**Table 2:** An example of a table derived from the output file produced by the Value Reader and Finding Classifier for a patient with neovascular age-related macular degeneration

Patient ID	12349876						
Visit	Report type	Laterality	Total macular volume (mm^3^)	Central macular thickness (μm)	…	Finding	Activity
2013-07-17	Unilateral macula: current only	Right	9.08	364	…	Subretinal fluid (+)	N/A
2013-08-27	Unilateral macula: current only	Right	8.21	251	…	Subretinal fluid (+)	Improvement
2013-10-10	Unilateral macula: current only	Right	8.16	218	…	Subretinal fluid (−)	Stationary
…	…	…	…	…	…	…	…
2016-06-02	Unilateral macula: current only	Right	8.34	236	…	Subretinal fluid (−)	Stationary
2016-08-19	Unilateral macula: current only	Right	8.29	238	…	Subretinal fluid (−)	Stationary
…	…	…	…	…	…	…	…
2019-08-29	Unilateral macula: current only	Right	8.05	189		Subretinal fluid (−)	Stationary

Longitudinal data with a total of 37 tests from July 2013 to August 2019

## Discussion

In this study, we extracted information from approximately 400,000 OCT report files. The overall accuracy of Value Reader was 99.67%. The results generated by Value Reader and Finding Classifier were stored in CSV files, which were subsequently imported into the OMOP CDM easily. We also plan to load cropped tomographic images into the PACS as soon as possible.

Several studies that used open-source or commercially available OCR software to extract text from optical coherence tomography have been described. [15–17] Aptel et al. used a commercial software, ABBYY FineReader v 9.0 (Avanquest Software, LaGarenne Colombes, France), and Demea et al. used NI Vision Assistant and NI Vision Development Module (National Instrument, Austin, Texas, USA) [15, 16]. Sood et al. [17] used Tesseract, an open-source OCR engine. The researchers used their OCR modules to extract only one or two types of research-specific data. They could not structure the data for general purposes. The development of customized and flexible software providing standardized, structured, and interoperable information from various ophthalmic examination devices has not been previously reported.

Report image files are simply an array of pixels unless a clinician manually reviews them. It is essential for clinicians to base decisions on comparisons of test results, however current report image files make this process difficult. The new software described in this paper enables the extraction of information from image files written in a non-standardized fashion and subsequently standardizes it in a structured CSV file. It can accommodate various types of image data and makes it easier for clinicians to interpret by organizing data. Structured information may be used in various ways; for example, an ophthalmologist may use it to interpret disease activity or progression by comparing measurement values or by referring to an automatically generated result. These features aid clinicians in decision-making without delays or errors. Processed data such as serial changes in macular thickness, macular volume, and disease activity of neovascular age-related macular degeneration in Fig. 2 can help ophthalmologists reduce misdiagnosis and determine appropriate timing of treatment. However, calibration among values may be considered in cases where the measurement values are different despite examining the same target. (e.g., devices from multiple vendors or upgraded measurement technique).

Deep learning has the potential to revolutionize medicine as demonstrated by a recently developed model with good performance for classification of pathologic findings [2]. In terms of accuracy, previous studies did not address the accuracy of their OCR modules [15–17]. However, there is a limit to improving accuracy by using commercial software or open-source library in our experience. For this reason, we developed an OCR engine using deep learning. It is possible to achieve the high accuracy required in the medical field by using deep learning for optical character recognition, since new data can raise its accuracy continuously. In addition, it is vital that these types of models are applicable to real-world practice and that they address unmet clinical needs such as trend analysis or prediction of future disease activity. In this report, we showed how several algorithms implemented in Value Reader and Finding Classifier modules with deep learning enabled a real-world clinician to make practical decisions by providing pathologic findings or evidence of disease activity.

Numerous clinical data registries containing sizeable real-world data sets have recently been established [18]. In addition, OMOP CDM that allows systematic analysis by transforming and integrating clinical data into standardized formats are emerging as one of the most important approaches in medical research [19, 20]. If the large amount of data held in millions of report image files could be extracted and entered into the databases mentioned above using our newly developed software, the resulting datasets might become some of the most powerful clinical tools available worldwide. The reliability and reproducibility of studies can also be improved tremendously because researchers can easily build databases containing millions of data in standardized form with this software. We completed the data extraction from OCT reports and loaded them into the OMOP CDM at our hospital. However, cropped tomographic images have not yet been incorporated into the PACS due to hospital policy. As with other medical images, encapsulated tomographic images in the DICOM format will eventually be uploaded into the PACS. This process will provide a structure within the PACS enabling analysis of multi-modal images, and will expand the base for imaging studies.

Full-scale validation of the Finding Classifier was not performed since it was beyond the scope of our study. In addition, since permission by government authorities is required to employ the Finding Classifier in clinical practice, we only investigated its potential application. We are currently in the process of building a ground-truth dataset utilizing an in-house database and an increasing number of open databases, and therefore we expect that Finding Classifier will soon be made more reliable.

Our software has several limitations. First, it is difficult to convert characters with low resolution, although humans can read them relatively easily. Second, our software can organize data for large-scale retrospective research, but it lacks real-time function for practice. It is necessary to build a real-time extraction, transformation, and loading pipeline and to integrate it with an EHR system for clinical implementation. Third, we need to validate and further develop the Finding Classifier. Finally, other considerations such as calibration among the same type of measurements derived from different vendors are required.

## Conclusions

In conclusion, we have developed software consisting of Value Reader and Finding Classifier modules that enable access to standardized, structured, and interoperable results from various ophthalmic examination devices. This effort lays the groundwork for the construction of very powerful clinical medicine databases. In addition, the software program will also directly benefit clinicians and medical institutions since Spectralis OCT is one of the most widely used OCTs worldwide. Furthermore, we expect that this method may be adapted to function with OCT reports from other vendors, ophthalmic devices such as corneal topography, or other types of medical devices. For a clinically relevant software, real-time function and a more sophisticated model that can cover low-resolution or more irregular images should be developed in the future.

## Availability and requirements

- Project name: SNUBH_CharReadOCT
- Project home page: https://github.com/ophthal-cdm/ SNUBH_CharReadOCT
- Operating system(s): Platform independent
- Programming language: Python
- Other requirements: Python 2.7, Python modules—xlsxwriter, datetime, argparse, Image, opencv_python 3.4.2, torch 1.2.0, torchvision 0.4.0, keras 2.2.5, tensorflow 2.0 gpu
- License: GNU General Public License
- Any restrictions to use by non-academics: None

## Data Availability

The datasets generated and/or analyzed during the current study are not publicly available due to information content that could compromise the privacy of research participants.

## Abbreviations

OMOP: Observational medical outcomes partnership
CDM: Common data model
PACS: Picture archive communication system
DICOM: Digital imaging and communications in medicine
OCR: Optical character recognition
IRB: Institutional review board
OCT: Optical coherence tomography
RNFL: Retinal nerve fiber layer
HER: Electric health record
CSV: Comma-separated values
PDF: Portable data format
